# Synthesis and characterization of Co(II) porphyrin complex supported on chitosan/graphene oxide nanocomposite for efficient green oxidation and removal of Acid Orange 7 dye

**DOI:** 10.1038/s41598-024-65517-z

**Published:** 2024-07-24

**Authors:** Sahar H. El-Khalafy, Mahmoud T. Hassanein, Mohamed M. Alaskary, Nehal A. Salahuddin

**Affiliations:** Department of Chemistry, Faculty of Science, University of Tanta, Tanta, 31527 Egypt

**Keywords:** Acid orange 7 dye, Oxidative degradation, Nanocomposite, Graphene oxide, Chitosan, Hydrogen peroxide, Chemistry, Materials science

## Abstract

Catalytic degradation of Acid Orange 7 (AO7) by hydrogen peroxide in an aqueous solution has been investigated using cobalt(II) complex of 5, 10, 15, 20 Tetrakis [4-(hydroxy)phenyl] porphyrin [Co(II) TPHPP] covalently supported chitosan/Graphene Oxide nanocomposite [Co(II) TPHPP]-Cs/GO, as highly efficient and recoverable heterogeneous catalyst. The structures and properties of [Co(II) TPHPP]-Cs/GO nanocomposite were characterized by techniques such as UV–Vis, FT-IR, SEM, EDX, TEM, and XRD. The oxidation reaction was followed by recording the UV–Vis spectra of the reaction mixture with time at λ_max_ = 485 nm. [Co(II) TPHPP]-Cs/GO nanocomposite demonstrated high catalytic activity and could decompose 94% of AO7 within 60 min. The factors that may influence the oxidation of Acid Orange 7, such as the effect of reaction temperature, pH, concentration of catalyst, Acid Orange 7, and hydrogen peroxide, have been studied. The results of total organic carbon analysis (TOC) showed 50% of dye mineralization under mild reaction conditions of AO7 (1.42 × 10^−4^M) with H_2_O_2_ (8 × 10^−2^M) in the presence of [Co(II) TPHPP]-Cs/GO nanocomposite (15 × 10^−3^ g/ml) and pH = 9 at 40 °C. The reuse and stability of the nanocomposite were examined and remarkably, even after six cycles of reuse, there was no significant degradation or deactivation of the recycled catalyst. Residual organic compounds in the reaction mixture were identified by using GC–MS analyses. The radical scavenging measurements and photoluminescence probing technology of disodium salt of terephthalic acid indicated the formation of the hydroxyl radical as the reactive oxygen species in the [Co(II) TPHPP]-Cs/GO nanocomposite/H_2_O_2_ system. A mechanism for the oxidation reaction has been discussed.

## Introduction

The chemical industry is of great importance in terms of its impact on the environment. The waste waters from this industry are generally strong and may contain toxic pollutants^1–3^. Acid Orange 7 (AO7) is located in the group of azo dyes that contain one or several auxochromes and chromophores in its molecular structure as an azo bond –N=N–^4,5^. The produced dye wastewaters in textile industries are usually toxic, non-biodegradable, and resistant to light and oxidizing agents^6,7^.

Many treatment strategies have been investigated and used to eliminate such organic contaminants from water involving physical, biological, and chemical treatments^8–16^. Recent progress in the removal of organics from wastewaters has led to the development of advanced oxidation processes (AOPs), which are in short based on the generation of extremely reactive species like hydroxyl and per hydroxyl radicals (HO^•^ and HO_2_^•^ respectively)^17^. Among them, the oxidation using Fenton’s reagent has proved to be a promising and attractive treatment method for the effective degradation of dyes, as well as for the destruction of many hazardous organic pollutants^18–21^.

Metalloporphyrin’s are known as efficient oxidation catalysts that have been successfully used in homogeneous catalysis, more active and more soluble in both organic and aqueous media. However, the self-decomposition such as dimerization and chemical routes to material oxidation often limited the application of metalloporphyrin’s^22,23^. To obtain more stable and versatile structures, metalloporphyrin molecules are often immobilized onto supported materials (chitosan, Graphene Oxide, zeolites, hydrogels, microparticles, etc.), which has led to remarkable activity hybrid materials with high stability for easy removal and reuse^24–36^.

Chitosan (Cs) is an environmentally friendly and renewable natural biopolymer with outstanding properties of non-toxic, biocompatible, and biodegradable. However, the low mechanical properties of chitosan restrict its use in a wide-range application^37–39^. Graphene oxide (GO), produced from natural graphite via a chemical exfoliation method, possesses abundant oxygen-containing functional groups (carboxyl and epoxy groups), Graphene Oxide (GO) has a high specific area, greater mechanical strength, and superior thermal stability as a two-dimensional material^40^. Nanocomposite technology using fillers such as Graphene Oxide is dispersed at low loading in the chitosan matrix as maximal mechanical enhancement and reinforcing agent for the polymer matrix because of their unique structure and properties^41^.

Hydrogen peroxide is considered a powerful, environmentally clean, and main oxidative intermediates for the degradation of various organic pollutants^42^.

**Figure 1:**
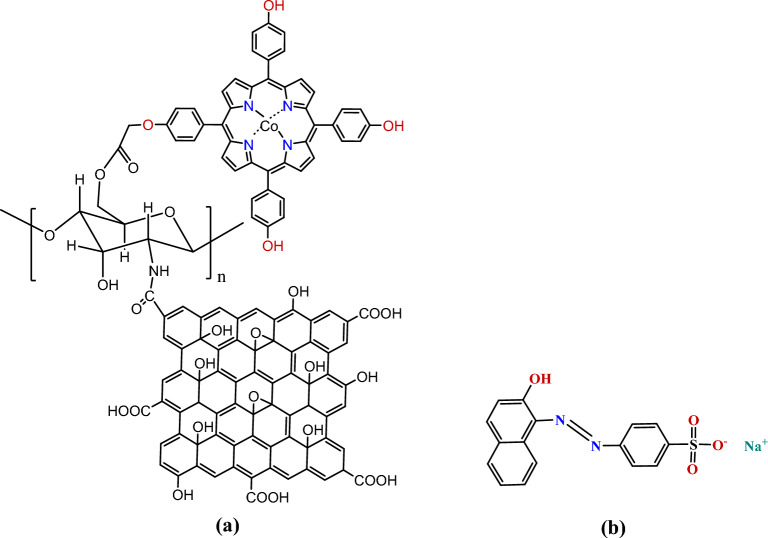
(**a**) [Co(II) TPHPP]-Chitosan/Graphene Oxide nanocomposite, (**b**) Acid Orange 7 (AO7).

We report in the present work the use of an efficient oxidation system based on hydrogen peroxide activated by the cobalt (II) porphyrin complex [Co(II) TPHPP] supported on modified chitosan/Graphene Oxide nanocomposite as a catalyst to carry out the degradation of Acid Orange 7 (AO7) in an aqueous solution (Fig. 1).

## Experimental section

### Materials and reagents

Pyrrole (97%) was distilled before use, graphite powder (99%), and Acid Orange 7 dye (dye content ≥ 85%) was purchased from (Merck Darmstadt, Germany). 4-hydroxybenzaldehyde (98%) and propionic acid (99.5%), Chitosan medium Mwt with high purity (Mwt 150,000 g/mol, degree of deacetylation 81%), *N*,*N*-dimethylformamide (DMF) (99.8%), and cobalt (II) chloride hexahydrate (≥ 97%) were obtained from (Sigma-Aldrich). Hydrazine hydrate (80%), chloroacetyl chloride (> 99%), phthalic anhydride (98%), and triethylamine (99%) were obtained from (Alpha-Chemika, India). (El-Nasr Pharmaceutical Chemicals, Egypt) supplied chloroform (99.4%), methylene chloride (99.9%), methanol (99.9%), concentrated sulfuric acid (99%), phosphoric acid (99%), sodium carbonate, potassium permanganate, and hydrogen peroxide (30%, as an oxidant), and its initial concentration was determined using potassium permanganate^43^. Silica gel (60–120 mesh) was purchased from (Fisher Co., New Jersey, USA).

### Instrumental measurements

On a Bruker Avance II spectrometer operating at 400 MHZ, 1HNMR spectra was measured in the presence of CDCl_3_ and the chemical shifts were provided. The UV–visible spectra were measured by using a UV-1800 UV–visible scanning spectrophotometer (SHIMADZU, Kyoto, Japan) in the range of 200–600 nm. FTIR was performed using (JASCO FT-IR-4100, Japan) in the range 400–4000 cm^−1^ utilizing liquid samples of semi-solid polymers or KBr pellets for powdered polymers with chloroform as the solvent. Analyzing the crystallography structure was done using an X-ray powder diffractometer (APD 2000 pro-Italy). The experiment utilized Cu-K radiation, which has a wavelength of 1.5406 Å. The angle range for the scan was set from 5 to 90, with a scanning rate of 0.05/s at 45 kV and 0.8 mA. The pH of the medium was adjusted using a pH bench meter (AD1030, Adwa, Hungary). Scanning electron microscope (SEM) and Energy-dispersive X-ray spectroscopy (EDX) analysis at 10 kV to detect the existence of elements within the nanocomposite using (JSM-IT200 In Touch Scope™ Scanning Electron Microscope). A transmission electron microscope (TEM) JEM-1400Plus Electron Microscope was used to examine the sample. After undergoing ultrasonic treatment, a small amount of the sample was delicately placed onto a copper grid. The solution was allowed to air dry at room temperature, and any excess liquid was removed by gently blotting it with a delicate piece of cloth. Filter paper by Whatman. GC-MASS analysis was performed using (Thermo Scientific ISQ single quadrupole gas chromatography-mass spectrometry (GC–MS) instrument).

### Synthesis of the catalyst

#### Synthesis of 5, 10, 15, 20 Tetrakis [4-(hydroxy) phenyl] porphyrin [TPHPP]

5,10,15,20 Tetrakis [4-(hydroxyl) phenyl] porphyrin [TPHPP] was prepared and purified according to the reported method^44^.

^1^HNMR (400 MHz, in DMSO): δ (ppm) 10.03 (s,4H, OH), 8.9 (s, 8H, βH), 8.03 (d, 8H, ArH), 7.24 (d, 8H, ArH), − 2.84 (s, 2H, NH).

UV–Vis (CHCl_3_) λ_max_: 416, 516, 554, 592 and 649 nm.

IR (ν, cm^−1^): 3423 ν(O−H,N–H), 1469 ν(C=N), 808, ν(macrocycle ring’s N–H out of plane bending vibration), 1599 ν(N−H bending) ,1228 ν(C−N), 1001 ν(Co−N).

#### Synthesis of Cobalt (II) complex of 5, 10, 15, 20 Tetrakis [4-(hydroxy) phenyl] porphyrin [Co(II) TPHPP]

Co(II) porphyrin complex was obtained through [TPHPP] (200 mg;0.294 mmol) and CoCl_2_·6H_2_O (58 mg; 0.294 mmol) was dissolved in methanol (20 mL), and refluxed for 4 h, then distilled water (60 mL) was added, and the methanol was evaporated under vacuum. The solution was cooled, and the purple crystalline product was obtained (0.28 mmol)^45^.

^1^HNMR (400 MHz, in DMSO): δ (ppm) 10.03 (s,4H, OH), 9.13 (s, 8H, βH), 7.9 (d, 8H, ArH), 7.25 (d, 8H, ArH).

UV–Vis (CHCl_3_) λ_max_: 448, 544, 581 nm.

IR (ν, cm^−1^): 3423 ν(O−H) become broad and slightly shifted, and a new band around 1001 ν(Co−N).

#### Phthaloylation of chitosan

Chitosan (1 g) was heated with excess phthalic anhydride (2.7 g) in 40 ml DMF containing 5% (v/v) of water to give phthaloylation (PHCS) according to the reported method^46^.

IR (ν, cm^−1^): 3422 ν(O−H), 1715, 1759 ν(C=O), 869, 724 ν(C–H, Ph), 1655 ν(C=C).

#### Grafting chloro-acetyl chloride on *N*-phthaloyl chitosan

Chloro-acetylation of *N*-phthaloyl chitosan was obtained according to the reported method^47^.

IR (ν, cm^−1^): 3477 ν(O−H), 2958 ν(C−H, CH_2_), 1717, 1763 ν(C=O), 1529 ν(C−H, ph), 1375, 1153 ν(C−O−C), 1064 ν(C−O), 717 ν(C–H, Ph).

#### Preparation of *N*-phthaloyl chitosan supported of [Co(II) TPHPP]

Grafting of [Co(II) TPHPP] complex on *N*-phthaloyl chitosan was done as a reported method^48^**,** with some modification briefly (0.01 mmol) cobalt porphyrin complex in DMF (30 mL) was added to a solution of *N*-phthaloyl chitosan (1.0 g) and anhydrous potassium carbonate (0.5 g).

IR (ν, cm^−1^): 3432 ν(O−H), 1720, 1661 ν(C=O), 1388 ν(C−N), 1001 ν(Co−N).

#### Deprotection of phthaloyl group

*N*-Phthaloyl chitosan/[Co(II) TPHPP] (1.0 g) was incubated with 2 ml hydrazine monohydrate at 100 °C for 2 h to deprotect the phthaloyl group. the product was collected, washed several times with water and ethanol, and then dried overnight in a vacuum to obtain [Co(II) TPHPP]–chitosan^49^.

IR (ν, cm^−1^): 3424 ν(O−H), 1656 ν(C=O), 1379 ν(C−N), 1001 ν(Co−N).

#### Preparation of [Co(II) TPHPP] supported on to modified chitosan/graphene oxide nanocomposite

Graphene Oxide was synthesized via the modified Hummer’s method^50^. Then, [Co(II) TPHPP]**-**Cs/GO nanocomposite [(4:1) ratio of [Co(II) TPHPP]**-**CS:GO] was synthesized via a facile two-step method according to the reported method^51^.

IR(ν, cm^−1^): 3398 ν(O−H), 1632 ν(C=O), 1431 ν(N−H deformation vibration), 1001 ν(Co−N).

#### Catalytic degradation of AO7 using [Co(II) TPHPP]-Cs/GO nanocomposite by H_2_O_2_

Acid orange 7 dye degradation in aqueous media using [Co(II) TPHPP]-Cs/GO nanocomposite as a catalyst with H_2_O_2_ was performed as follows: (15 × 10^−3^ g/ml) of [Co(II) TPHPP]-Cs/GO nanocomposite was put in a flask with 10 mL of (1.42 × 10^−4^ M) of AO7 dye aqueous solution with pH was adjusted to 9.0 by using borax and HCl buffer mixture, and the solution was stirred at 40 °C. (8 × 10^−2^M) H_2_O_2_ was added to start the reaction, and UV–visible spectroscopy was used to follow the degradation rate. At desired time intervals, 3 mL aliquots were taken out of the reaction flask and subjected to analysis. The UV of AO7 was calculated at wavelengths of 485 nm. The aliquots were then added back to the reaction flask. Pseudo-first order relation was applied to fit the results from destruction results:

1 $$ \ln ({{\text{A}}_{\text{o}}}/{{\text{A}}_{\text{t}}}) = {{\text{k}}_{{\text{obs}}}} \times {\text{t}} $$

where A_o_ is the dye’s initial absorbance (at t = 0 min), A_t_ is that absorbance at time = t, and K_obs_ (min^−1^) is the observed rate constant calculated from the slope of the linear plot of ln (A_o_/A_t_) vs time.

#### Recovery and recycling of catalyst

[Co(II) TPHPP]-Cs/GO nanocomposite were recycled. After the completeness of the reaction, the supported catalyst was removed from the reaction media by simple filtration, and they were reused for subsequent experiments after being washed with distilled water. Then directly used in the next degradation reaction^52^. Successive catalytic degradation experiments under optimum conditions were achieved to investigate the recycling ability of the catalyst for at least 6 cycles. For these tests, the same sample was utilized. The following parameters were set for the experiment: 10 mL solution volume; initial 1.42 × 10^−4^ M dye concentration, 15 × 10^−3^ g/mL of catalyst dosage, 8 × 10^−2^ M H_2_O_2_ concentration at 40 °C and reaction time: 60 min. The degradation efficiency was investigated according to:

2 $$ {\text{Degradation}}\,{\text{efficiency }}\% = [({{\text{A}}_{\text{o}}} - {{\text{A}}_{\text{t}}})/{{\text{A}}_{\text{o}}}] \times 100 $$

A_o_ is the absorbance of dye at zero time, and A_t_ is the absorbance of dye at time “t,” at *λ*_max_ = 485 nm.

## Results and discussion

**Figure 2:**
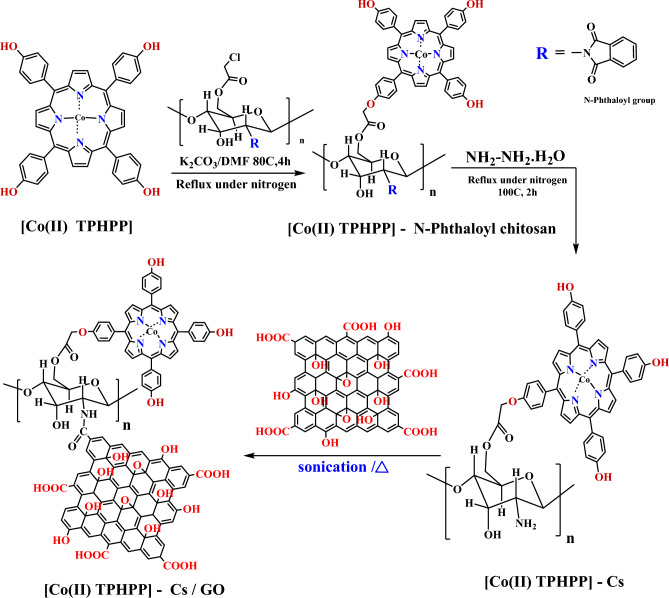
The schematic illustration of the prepared [Co(II) TPHPP]-Cs/GO nanocomposite.

5,10,15,20 Tetrakis [4-(hydroxyl) phenyl] porphyrin [TPHPP] was prepared by condensing pyrrole and 4-hydroxybenzaldehyde in propionic acid^44^. Metalation of porphyrin with CoCl_2_·6H_2_O in DMF form [Co(II) TPHPP]^45^. Simultaneously the amino group of biopolymer chitosan was protected by using phthalic anhydride forming *N*-Phthaloyl chitosan^46^, which chloroacetylated using chloroacetyl chloride in the presence of triethyl amine forming Chloroacetylated *N*-phthaloyl chitosan^46^. [Co(II) TPHPP] covalently bonded to (chlorocetylated *N*-phthaloyl chitosan) by refluxing in the presence of potassium carbonate^48^. Then, the protected (phthaloyl group) was removed using Hydrazine monohydrate^49^. Finally, Chitosan/[Co(II) TPHPP] mixed, vigorously stirring with Graphene Oxide suspended solution to form [Co(II) TPHPP]-Cs/GO nanocomposite^51^ as shown in (Supplementary Figs. S1–S7) Fig. 2.

### Structural characterizations and morphologies analysis

#### FT-IR analysis

**Figure 3:**
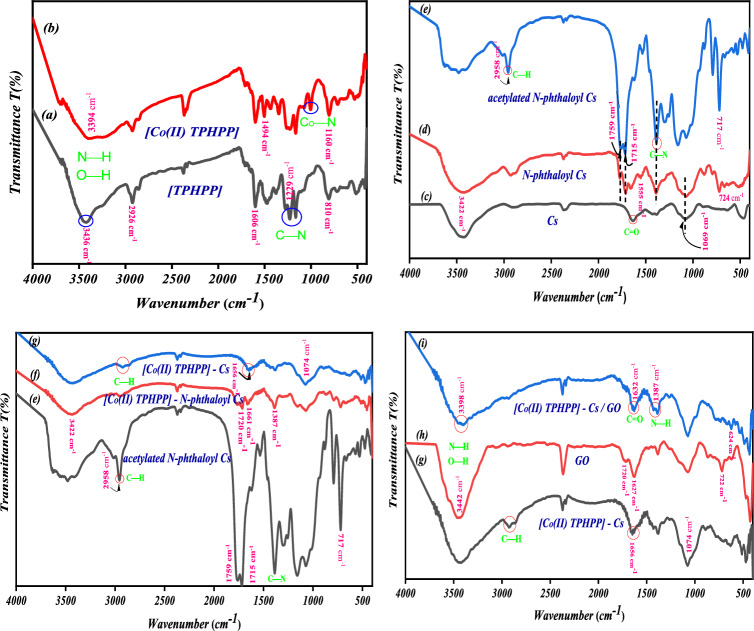
FT IR spectra of (**a**) [TPHPP], (**b**) [Co(II) TPHPP], (**c**) Chitosan, (**d**) *N*-Phthaloyl Chitosan, (**e**) acetylated *N*-Phthaloyl Chitosan, (**f**) [Co(II) TPHPP]-*N*-Phthaloyl Chitosan, (**g**) [Co(II) TPHPP]-Chitosan, (**h**) Graphene oxide, (**i**) [Co(II) TPHPP]-Cs/GO nanocomposite.

The FTIR spectrum of THPP is consistent with its chemical structure as shown in (Fig. 3a). The stretching vibrations of O−H and N−H groups showed a broad band at ν 3423 cm^−1^. Additionally, the peaks at ν 1228 and 1169 cm^−1^ were attributed to C−N stretching vibrations of amine groups, while peaks at ν 1599 and 1469 cm^−1^ were assigned to N–H bending and C=N vibrations, respectively. Finally, the peak at ν 808 cm^−1^ was referred to as the macrocycle ring’s N–H out-of-plane bending vibration. In the [Co(II) THPP] spectrum (Fig. 3b), the peak at ν 3423 cm^−1^ became broad and slightly shifted to 3420 cm^−1^. The new peak at ν 1001 cm^−1^ has appeared, which is characteristic absorption of the Co–N(equatorial) bond formed in [Co(II) THPP]^24,53^.

The spectrum of Cs (Fig. 3c) shows a broad band at ν 3424 cm^−1^, which is the region where the stretching vibration of O−H and N−H groups are situated due to intermolecular H-bond. The absorption bands at ν 1634 cm^−1^ and 1389 cm^−1^ correspond to the C=O stretching vibration (amide band) and C−N stretching vibration in amide. And band at ν 1069 cm^−1^ corresponds to C−O stretching vibration. Unlike chitosan, the FT IR spectrum of *N*-phthaloyl chitosan (Fig. 3d) exhibits characteristic peaks at ν 1715, 1759 corresponding to C=O of the phthalimide group. And another peak at ν 1555 and 724cm^−1^ for C=C stretching vibration and C−H bending vibration of an aromatic ring^54^.

For the chloroacetylated of *N*-phthaloyl chitosan (Fig. 3e), a new signal around ν 2958 cm^−1^ (methylene of the chloroacetyl group) was found to correlate with the decrease of the signal around ν 3424 cm^−1^ (hydroxyl groups). Also, a new peak at ν 717 cm^−1^ confirmed the presence of C–Cl in the chloroacetyl group^55–57^.

For [Co(II) TPHPP] supported on *N*-Phthaloyl chitosan spectrum (Fig. 3f), the broad peak around at ν 3486 cm^−1^ was slightly shifted to ν 3432 cm^−1^, and the intensity was diminished which related to vibration of O−H and N−H groups. Also, the band at ν 1759 and 1715 cm^−1^ was shifted at ν 1720 and 1661 cm^−1^, and the significant peak at ν 717 cm^−1^ which is related to C–Cl in the chloroacetyl group disappeared due to attaching [Co(II) THPP] **1** to *N*-phthaloyl chitosan. This proves that the [Co(II) THPP] **1** molecules were covalently bonded to *N*-phthaloyl chitosan. As seen, signature bands for [Co(II) THPP] did not appear anywhere in the spectrum of [Co(II) THPP]–*N*-phthaloyl chitosan due to the overlap of the spectra of *N*-phthaloyl chitosan and [Co(II) THPP] in the entire infrared region^58^.

FT-IR spectrum for the protected group (phthaloyl group) removed (Fig. 3g), chitosan/Co-porphyrin show disappearing characteristic peaks at ν 1720,1661,717 cm^−1^ correspond to C=O of phthalimide group and C−H bending vibration of aromatic ring. Another peak appeared at ν 1656 cm^−1^ this evidence removed the protected group (phthalic anhydride)^59^.

The spectrum of GO shown in (Fig. 3h), exhibited a broad band at 3442 cm^−1^ for O–H groups, and two characteristic bands for C=O and C=C at 1720, and 1627 cm^−1^, respectively. Furthermore, the other peaks corresponding to C–OH and C–O stretching vibration appeared at 1387 and 1074 cm^−1^, respectively. These characteristic peaks indicated that the GO nanosheets were successfully synthesized^60^.

The final FT-IR spectrum of [Co(II) TPHPP]-Cs/GO nanocomposite (Fig. 3i), starching vibration of C=O in amide downshift from ν 1651 to 1632 cm^−1^, and NH_2_ deformation vibration downshift from ν 1438 to 1431 cm^−1^. indicate an amide bond between NH_2_, and COOH, This evidence for the formation of an amide bond (–NHCO–)^61^.

#### XRD structural characterization

**Figure 4:**
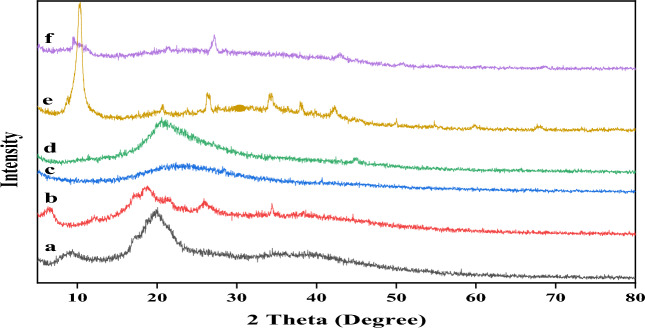
X-ray pattern of (**a**) Chitosan, (**b**) *N*-Phthaloyl Chitosan, (**c**) *N*-Phthaloyl Chitosan-[Co(II) TPHPP], (**d**) Chitosan-[Co(II) TPHPP], (**e**) Graphene Oxide, (**f**) [Co(II) TPHPP]-Cs/GO nanocomposite.

The X-ray powder diffraction pattern was illustrated in Fig. 4 where (Fig. 4a) the original chitosan shows a weak peak at 2θ = 9.60° and a more intense peak at 2θ = 20.22° that is caused by diffraction from the (020) and (110) planes of the crystalline lattice with interplane distances of 0.92 nm and 0.438 nm, respectively. It is worth noting that the corresponding degree of acetylation was 81% and its crystalline index was 55^62–65^.

*N*-phthaloyl chitosan (Fig. 4b) showed the disappearance of the peak at 2θ = 9.6° with the appearance of another peak at 6.42°, and the peak at 2θ = 20.22° was shifted to 2θ = 18.62°. This explains why the amount of hydrogen bonds decreased by blocking amino groups, leading to a typical diffraction pattern with lower crystallinity. It was reported that *N*-phthaloyl chitosan prepared in DMF/water showed a certain crystallinity, despite the introduction of the phthaloyl group; this also supports the uniform structure of the product. It is noteworthy that *N*-phthaloyl-chitosan exhibited certain crystallinity even though such a bulky substituent had been introduced^64^.

*N*-Phthaloyl chitosan-[Co(II) TPHPP] shows a more broadening peak at around 2θ = 22.8° (Fig. 4c). The addition of [Co(II) TPHPP] to *N*-Phthaloyl Chitosan reacts with the hydroxyl group, further decreasing crystallinity where no more hydrogen bonding exists^66^.

Figure 4d shows that the deprotection of the amino group of chitosan (phthaloyl group) shows a characteristic peak at 2θ = 20.81°, which indicates a higher crystallinity of this compound.

Figure 4e show a sharp peak at 2θ = 10.32°, which confirms that graphite powder was oxidized well, utilizing concentrated acids and KMnO_4_ to obtain GO. After modifying GO with modified [Co(II) TPHPP]-Cs, the significant peak of GO at 2θ = 10.32° shifted to a smaller angle and appeared at 2θ = 9.60° as shown in (Fig. 4f). This shift is due to the intercalation of GO by modified metalloporphyrin-chitosan chains. The two peaks of Cs are not observed in the XRD patterns of GO-Cs nanocomposite, indicating that [Co(II) TPHPP]-Cs chains are well intercalated amongst the GO sheets. This confirms the good attachment of [Co(II) TPHPP]-Cs to GO layers^67,68^.

#### SEM and TEM analysis

One of the most important factors affecting the heterogeneous catalysis behavior is the morphological characteristics of their surface. Therefore, the interfacial interactions between [Co(II) TPHPP]-Chitosan and Graphene Oxide can change the surface morphology of the obtained nanocomposite.

##### SEM and EDX technique

**Figure 5:**
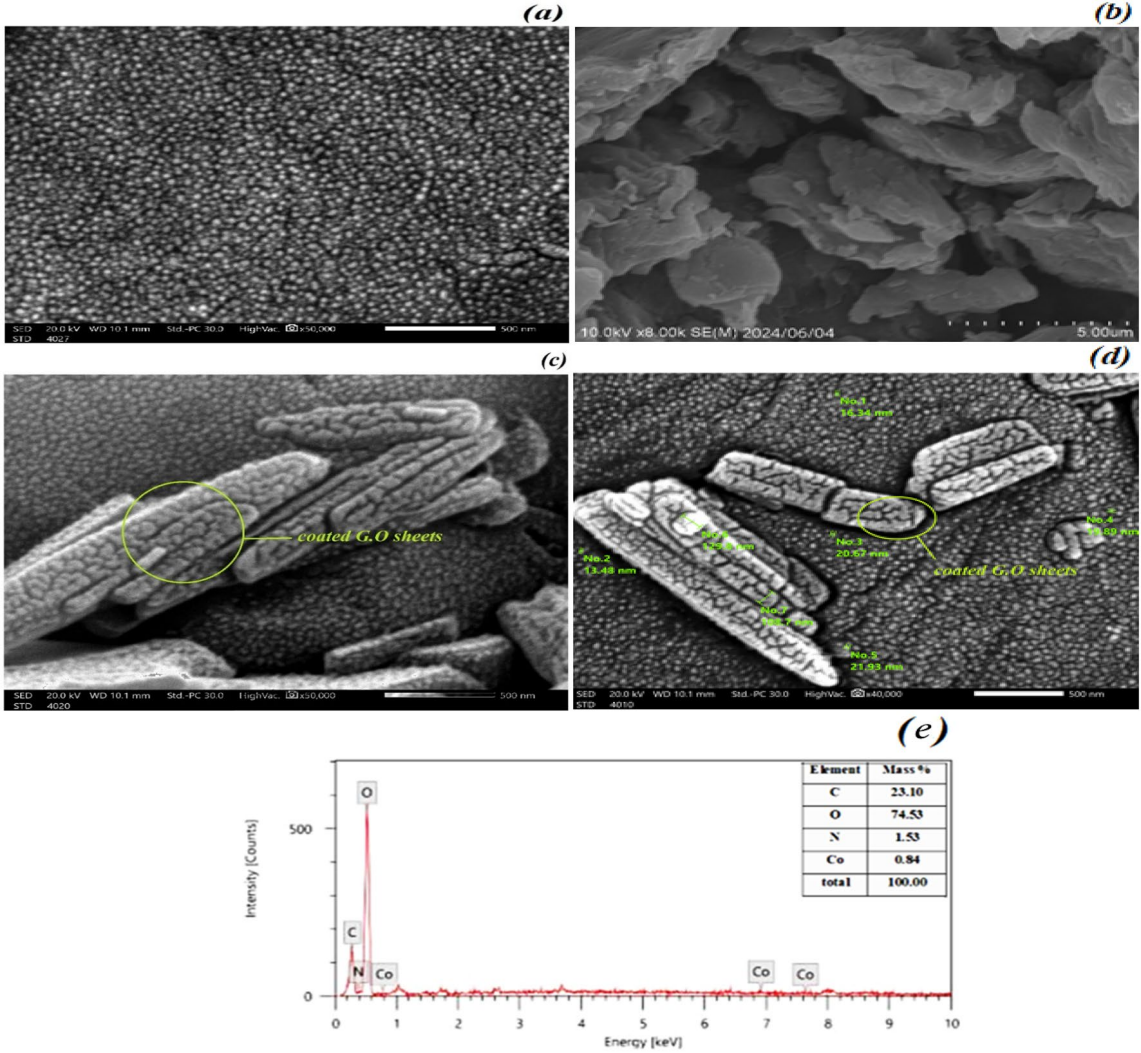
Scanning electron microscope (SEM) analysis of [Co(II) TPHPP]-Cs (**a**), Graphene Oxide (**b**), [Co(II) TPHPP]-Cs/GO nanocomposite (**c**,**d**), and (**e**) indicating the elements by EDX analysis.

SEM micrograph of the [Co(II) TPHPP] supported on chitosan before and after GO incorporation, where the uniform distribution of [Co(II) TPHPP]-chitosan network is displayed in (Fig. 5a). The typical morphology of GO is shown in (Fig. 5b), which has a smooth, flat, soft surface and many layers stacked on top of one another in a range of sizes and forms. Furthermore, as seen in (Fig. 5c,d), the irregularly shaped GO layers and their surface developed wrinkles as a result of being occupied by [Co(II) TPHPP]**-**Cs beads, where the [Co(II) TPHPP]-Cs beads bind the bunches of flake GO sheets, these bound transpire on the surface and from edges confirm the tightly bound with each other and illustrate the successful interaction between [Co(II) TPHPP]**-**Cs and the GO. Additionally, similar results were supported using the EDX analysis, which shows the presence of elements such as C, O, and N, Co, which indicate the combination between them forming [Co(II) TPHPP]**-**Cs/G’O nanocomposite in (Fig. 5e).

##### TEM analysis

**Figure 6:**
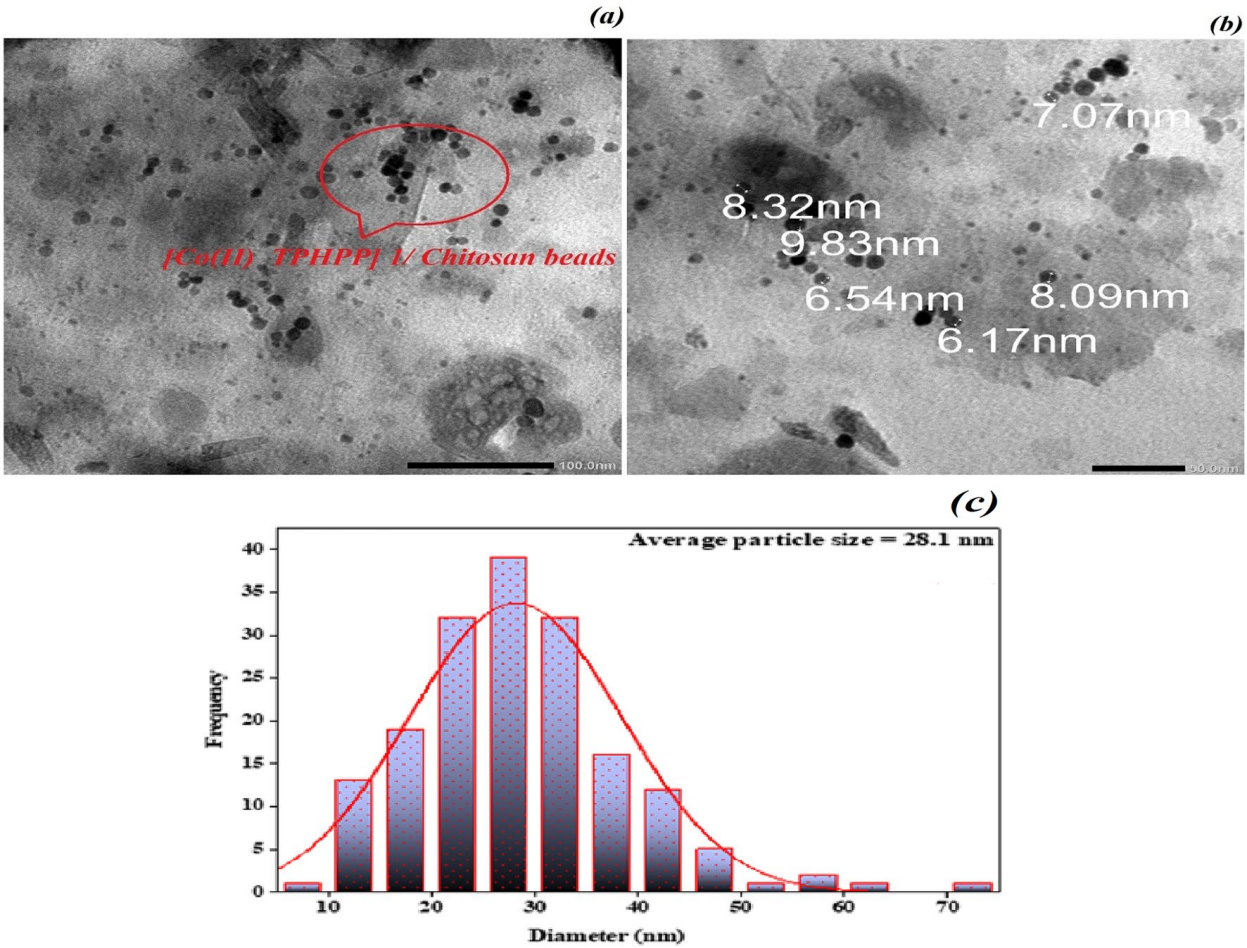
Transmission electron microscope (TEM) analysis of [Co(II) TPHPP]-Cs/GO nanocomposite (**a**,**b**), and (**c**) Histogram of particle size distribution curve of [Co(II) TPHPP]-Cs/GO nanocomposite from (TEM).

The TEM imaged in (Fig. 6a,b), shows the nanoparticles morphologies of [Co(II) TPHPP]**-**modified Chitosan are mostly spherical. Some of these beads spread and others aggregated within Graphene Oxide layers. The TEM image shows that the changes to the inner layers of GO are dramatic and clearly visible as some layers intercalated and others exfoliated. Where the bulk of GO sheets contain beads with wrinkles due to the dispersion of [Co(II) TPHPP]**-**chitosan networks on the GO sheets. Figure 6c shows the histogram of the particle size distribution curve of [Co(II) TPHPP]**-**Cs/GO nanocomposite where the irregular spherical shape of these beads and its average particle size (< 30 nm).

### Catalytic oxidation of AO7

**Figure 7:**
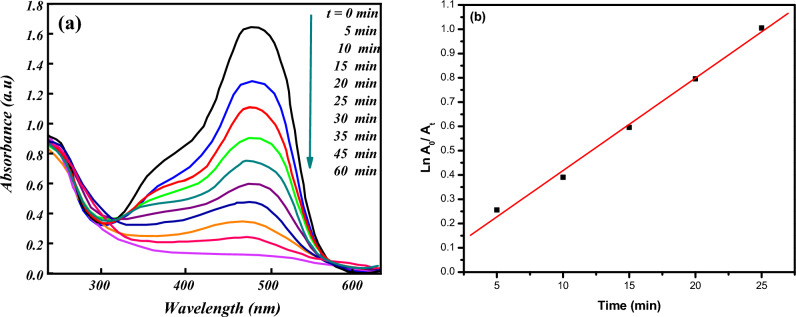
(**a**) Electronic absorption spectra during decolorization of AO7, (**b**) First-order plot for the degradation of AO7 with (k_obs_ = 0.236 min^−1^)_._ For reaction conditions: AO7 (1.42 × 10^−4^ M) with H_2_O_2_ (8 × 10^−2^ M) in the presence of [Co(II) TPHPP]-Cs/GO nanocomposite (15 × 10^−3^ g/mL) and pH = 9 at 40 °C.

The catalytic oxidation of (AO7) has been investigated using hydrogen peroxide as an oxidant and [Co(II) TPHPP]**-**Cs/GO nanocomposite as a catalyst in aqueous solution. The oxidation reaction was followed by recording the UV–Vis spectra of the reaction mixture with time at λ_max_ = 485 nm. (Fig. 7a) represents the collapse of the main absorbance band of AO7 at λ_max_ = 485 nm almost vanished, and the degradation percent of AO7 reached 94% within 60 min, this is due to the lost conjugation in the dye leading to colorless oxidation products. This reverberates an essential role of using H_2_O_2_ and [Co(II) TPHPP]**-**Cs/GO nanocomposite in the degradation of AO7. Figure 7b also shows the kinetic curve for the destruction of AO7, the plot of (lnA_0_/A_t_) against time presents a straight line, this indicates that the degradation of AO7 is considered a first-order rate kinetic^69,70^ and it could be simply described as ln A_0_/A_t_ = k_obs_ t.

Before studying the behavior of this supported catalyst, it is important to evaluate the AO7 decolorization process, i.e., whether AO7 removal occurs through adsorption, through a catalytic reaction, or both processes. For that reason, several runs were then performed. The first one was a blank, carried out to evaluate the ability of H_2_O_2_ to eliminate AO7 in aqueous solutions without the addition of the heterogeneous catalyst which shows that AO7 degradation due to hydrogen peroxide is almost negligible (Supplementary Fig. S8) which might be attributed to its low oxidation potential as compared to hydroxyl radicals.

To determine the influence of the adsorption processes experiment without, H_2_O_2_ and in the presence of [Co(II) TPHPP]-Cs/GO nanocomposite was carried out as shown in (Supplementary Fig. S9) and also carried out at different pH used (7,9 and 11) (Supplementary Fig. S10), which indicated no significant decolorization of dye, this evidence for the degradation pathway occurred through a catalytic reaction.

### Influence of the experimental conditions on oxidation of AO7

The factors that may influence the oxidation of AO7, such as the Influence of reaction pH, AO7, concentration of catalyst, hydrogen peroxide, and temperature have been investigated.

#### Influence of initial PH

**Figure 8:**
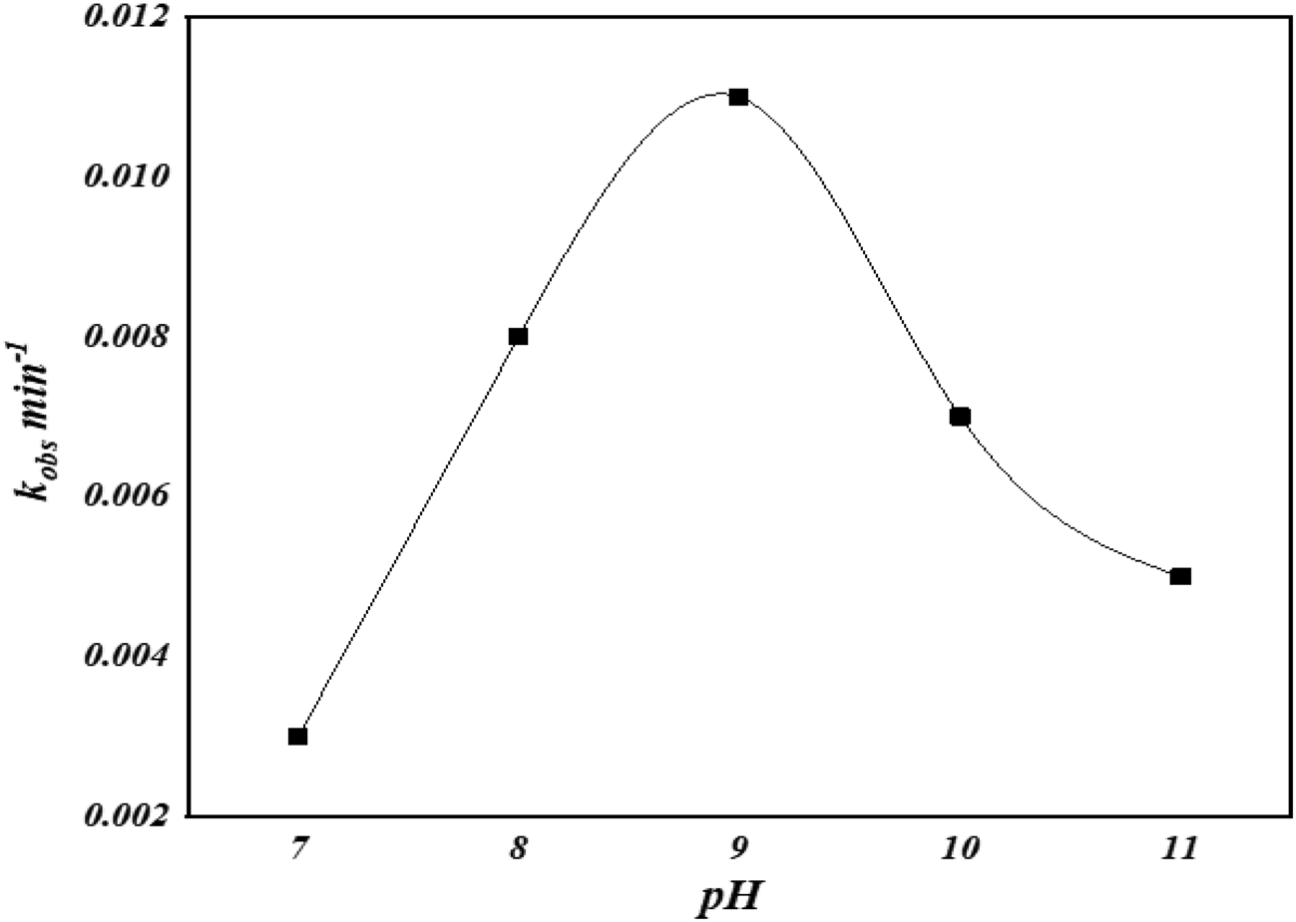
Effect of pH on the decolorization of AO7. The pH was adjusted to 8 and 9 using borax and HCl buffer mixture and the pH was adjusted to 10.0 using NaHCO_3_ and NaOH mixture. Phosphates were used to adjust the pH to 7 and 11.

The influence of pH on the oxidation reaction was investigated at a constant concentration of the dye (1.42 × 10^–4^ M), H_2_O_2_ (8 × 10^−2 ^M) as well as a fixed amount of [Co(II) TPHPP]-Cs/GO nanocomposite (15 × 10^–3^ g/mL) at 40 °C. The pH varied in the range of 7–11. Data illustrated in (Fig. 8) show that the decolorization of AO7 increased with an increase of pH and reached optimum at pH = 9.0^71,72^. The factors likely responsible for the decrease in the observed rate of constant k_obs_ at higher pH are the formation of hydroperoxide anion HO^−^_2_ in an alkaline medium, which reacts with the non-dissociated molecule of H_2_O_2_ according to reaction Eq. (3).

3 $$ {{\text{H}}_2}{{\text{O}}_2} + {\text{HO}}_2^- \to {{\text{H}}_2}{\text{O}} + {{\text{O}}_2} + {\text{H}}{{\text{O}}^- } $$

#### Influence of AO7 concentration

**Figure 9:**
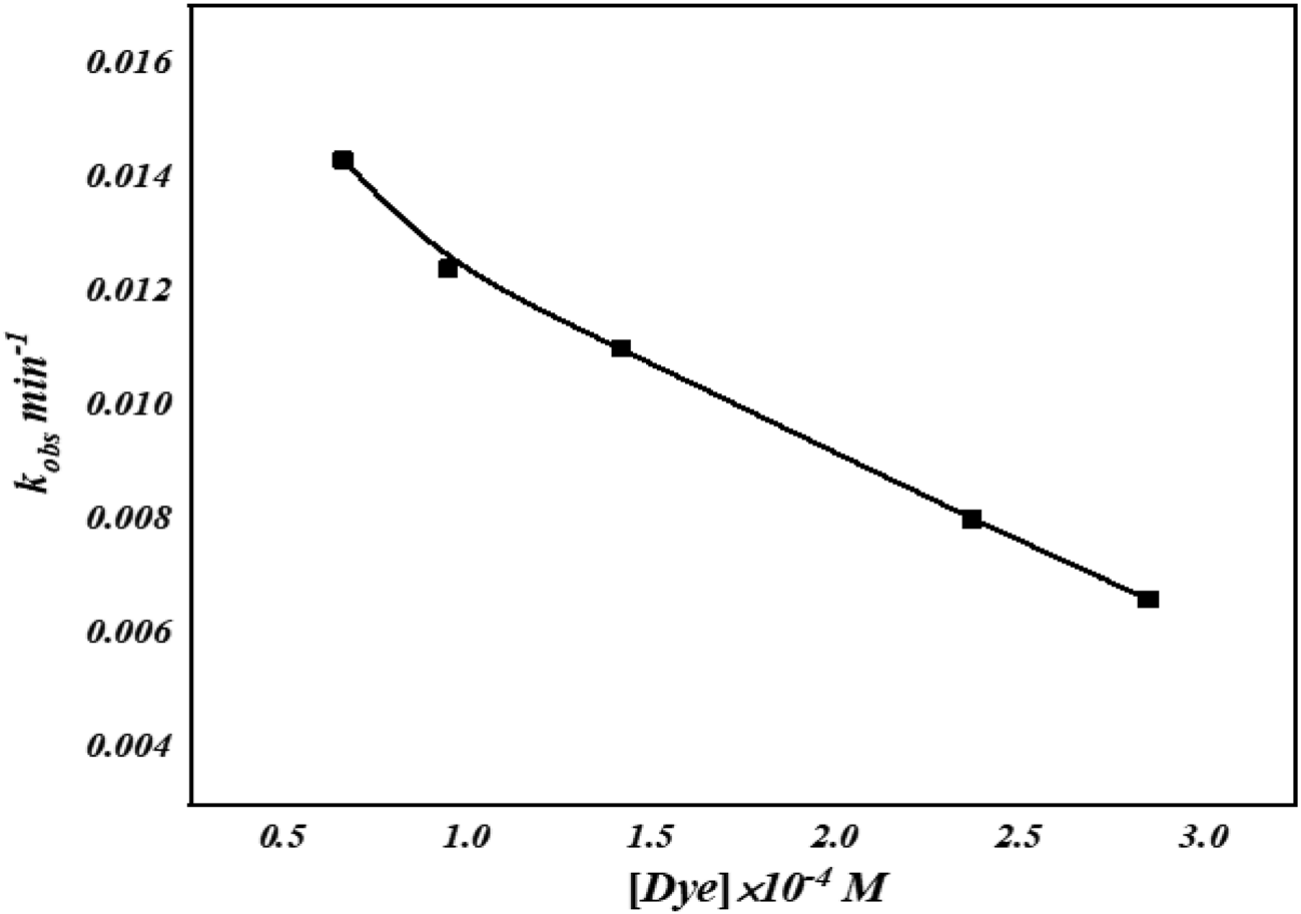
Effect of AO7 concentration on decolorization process.

The variation of the decolorization efficiency of the AO7 with H_2_O_2_ and [Co(II) TPHPP]-Cs / GO nanocomposite with varying the concentration of the dye from 6.65 × 10^−5^ M to 2.85 × 10^−4^ M. (Fig. 9) shows that the decolorization efficiency reduces with a further increase in dye concentration. This phenomenon may be attributed to the high concentration of AO7 molecules that may aggregate on the catalyst surface and inhibit contact between H_2_O_2_ and the catalyst, which reduces the number of hydroxyl radicals involved in the decolorizing process^72^. The molar ratio oxidant/dye is low (because the amount of hydrogen peroxide molecules initially present in the reaction is the same).

#### Influence of catalyst concentration

**Figure 10:**
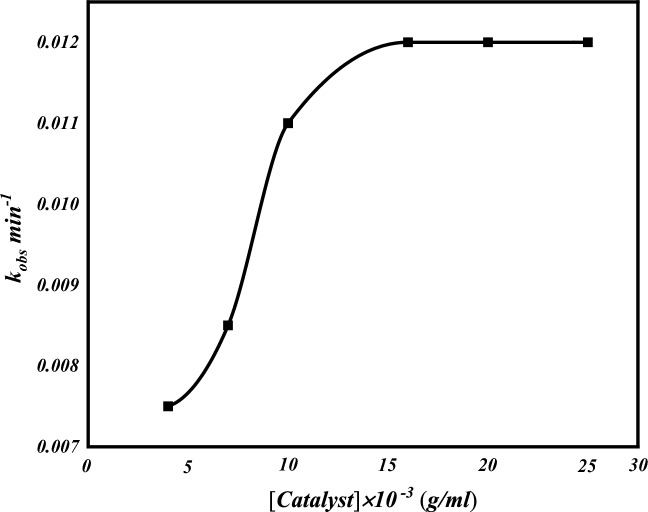
Effect of catalyst concentration on decolorization process.

Data illustrated in (Fig. 10) show that the rate of oxidation of AO7 increases with an increase of the catalyst concentration from 4 × 10^−3^ g/mL to 15 × 10^−3^ g/mL with all other reaction parameters fixed, at 40 °C. This may be attributed to the increase in the number of available active sites on the catalyst surface for H_2_O_2_ activation and the increased rate of formation of hydroxyl radicals^73^. After 15 × 10^−3^ g/mL concentration, adding more catalyst may not have a significant effect on the rate of reaction. This point is called steady-state concentration where no further increase in the rate of reaction, due to the limited number of attacking active species involved in the degradation process**, **k_obs_ remained almost constant. Thus, the optimum catalyst dosage^74^.

#### Influence of initial H_2_O_2_ concentration

**Figure 11:**
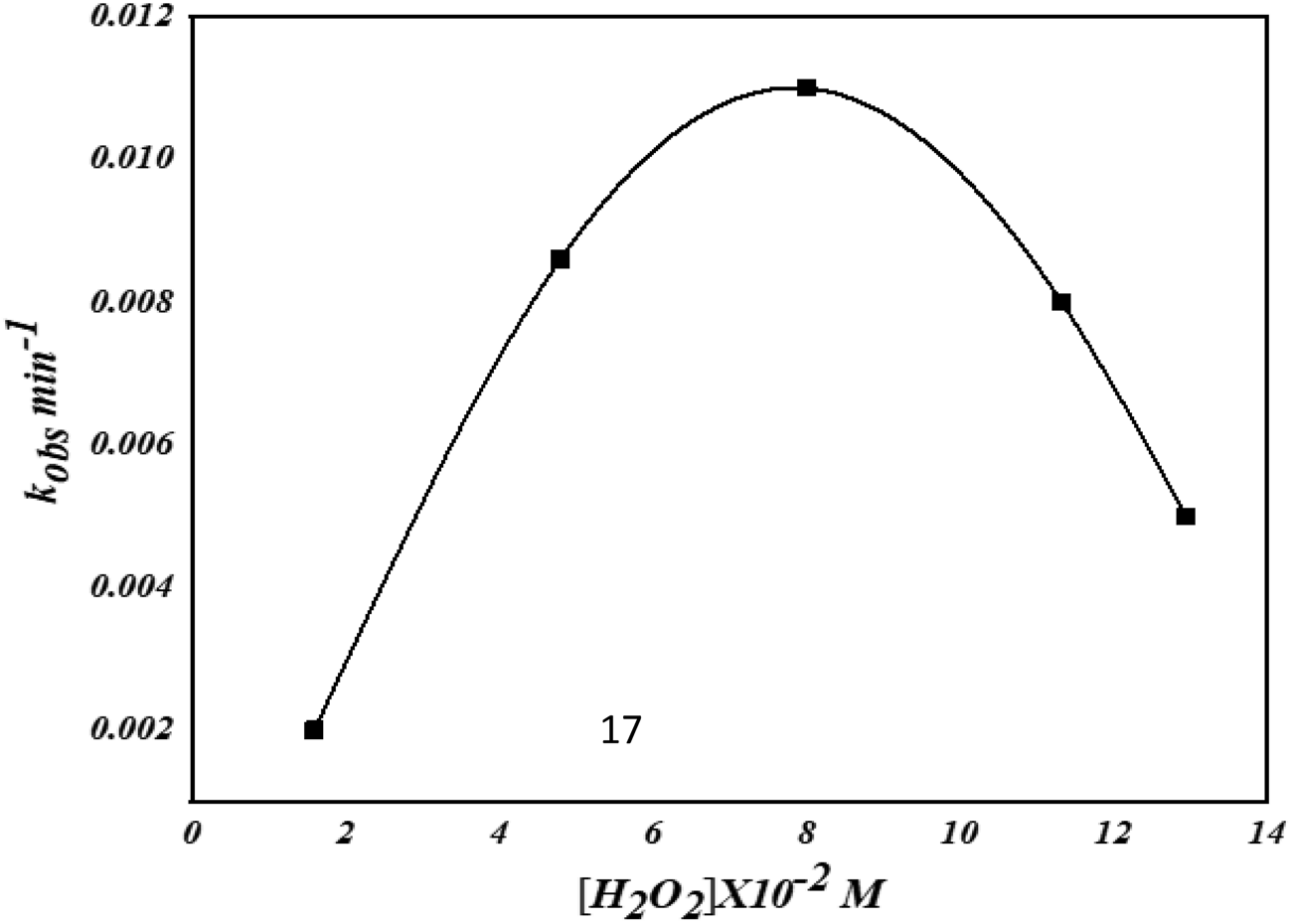
. Effect of concentration of H_2_O_2_ on the decolorization of AO7.

The effect of the concentration of H_2_O_2_ on the oxidation of AO7 was analyzed by varying its initial concentration between 1.6 × 10^−2^ M and 12.94 × 10^−2^ M at 40 °C (Fig. 11). Increasing H_2_O_2_ concentration from 1.6 × 10^−2^ M to 8.08 × 10^−2^ M increases the rate of decolorization of the dye AO7 because more ^•^OH radicals are formed. A further increase in the concentration of H_2_O_2_ partly inhibited the oxidation rate due to the well-known hydroxyl radicals scavenging effect. The undesirable reactions (4) and (5) compete with the destruction of the dye chromophore^72^. These reactions reduced the probability of attacking the AO7 molecules by hydroxyl radicals, which decreased the decolorization rate of the dye at a high concentration of H_2_O_2_.

4 $$ {{\text{H}}_2}{{\text{O}}_2} + {}^ \cdot {\text{OH}} \to {\text{HO}}_2^ \cdot  + {{\text{H}}_2}{\text{O}} $$

5 $$ {\text{HO}}_2^\cdot + {}^\cdot {\text{OH}} \to {{\text{H}}_2}{\text{O}} + {{\text{O}}_2}  $$

#### Influence of reaction temperature

**Figure 12:**
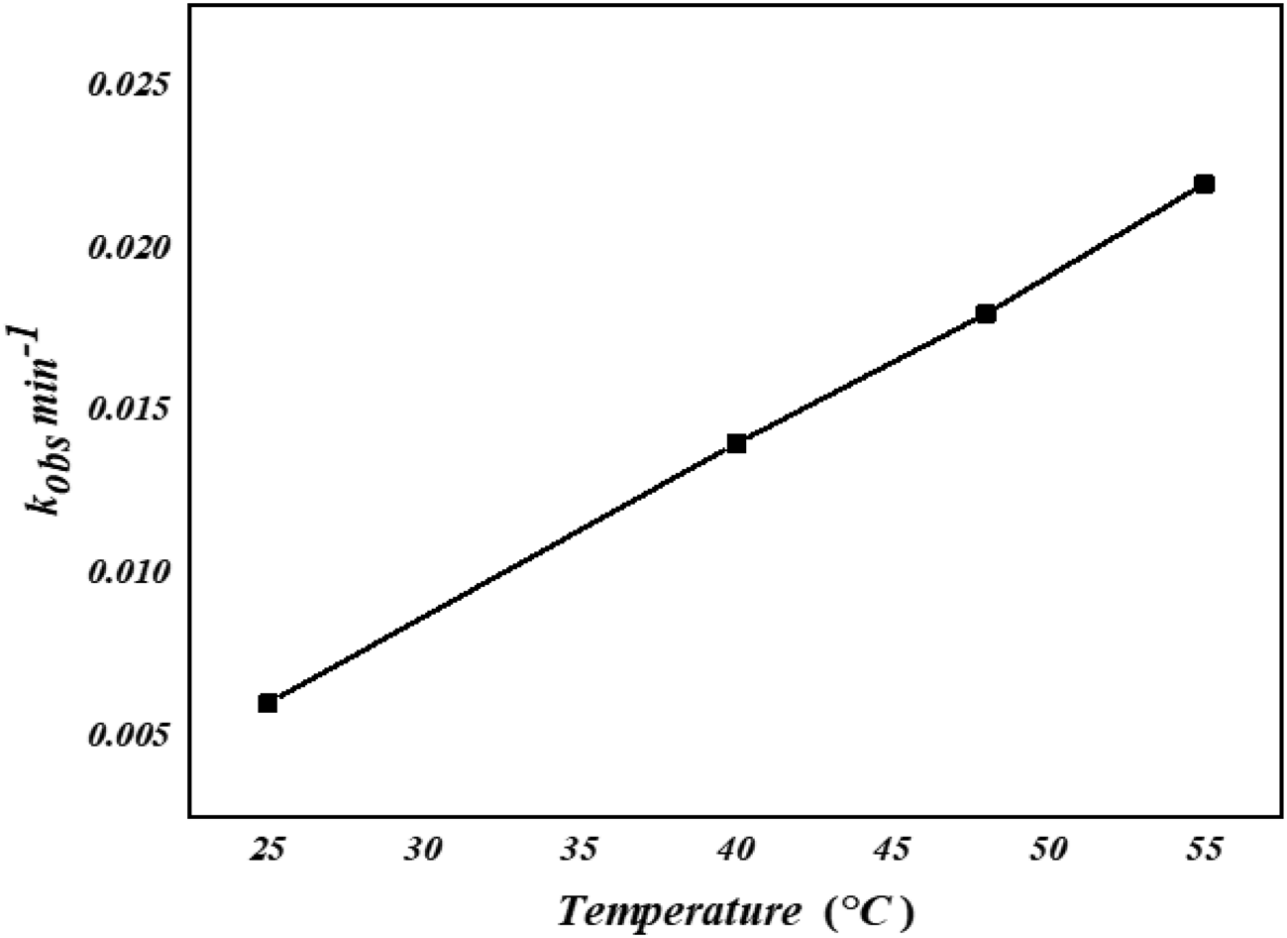
Effect of temperature on the decolorization of AO7.

The effect of temperature on the decolorization of AO7 with [Co(II)-TPHPP]**-**Cs/GO nanocomposite /H_2_O_2_ has been studied in the range of 25–55 °C. (Fig. 12) shows that the decolorization rate increased with an increase in reaction temperature. Increasing temperature led to a shorter time for decolorization of AO7.

Thermodynamic parameters for degradation AO7 such as Gibbs free energy (ΔG°), enthalpy (ΔH°), and entropy (ΔS°) describe the nature and type, and disorder of the system at the liquid–solid interface^75^.

Data illustrated in Table 1 where: a positive ∆G° value validates a non-spontaneous process under standard conditions, which means that the reaction still prefers reactants over products under the given conditions. Overall, a positive ΔG with a catalyst doesn’t necessarily mean an ineffective reaction. It just means the reaction leans toward the reactants, and the catalyst helps achieve equilibrium faster^76,77^. A positive ∆H° value is indicative of an endothermic, irreversible process, and the negative value of ΔS° revealed that the degree of the disorder decreased at the solid–liquid interface during the reaction and the positive E_a_ is an indication the system requires energy (increased temperature) to drive the process, therefore is termed endothermic^78^.

**Table 1:** Rate constant and activation parameters of catalytic degradation of AO7 by [Co(II) TPHPP] -Cs / GO nanocomposite with H_2_O_2_.

Temperature (°K)	K_obs_ (min^−1^)	ΔΓ^#^ (k J μοl^−1^)	Ea (k J mol^−1^)	ΔΗ^#^ (k J μοl^−1^)	ΔΣ^#^ (J mοl^−1^ Κ^−1^)
289	0.006	85.79			
313	0.011	88.45	35.5	35.5	− 1.77 × 10^2^
321	0.013	89.87			
328	0.025	91.11			

### Mineralization and proposed degradation pathway of AO7

Total organic carbon analysis (TOC) is an important method for evaluating the mineralization of the oxidative degradation reactions of dyes. Oxidation of AO7 under standard reaction conditions at 40 °C showed after 60 min TOC removal of 50% indicating incomplete mineralization of the dye to CO_2_ and H_2_O. However, raising the reaction temperature to 55 °C mineralization of the dye increased and the mineralization of AO7 also enhanced with a higher concentration of catalyst up to 15 × 10^–3^ g/mL^79–82^.

To further evaluate the efficiency of the catalytic [Co(II) TPHPP]-Cs/GO nanocomposite with an H_2_O_2_ system, Gas chromatography-mass spectrometry (GC–MS) analysis, was employed to study the degradation behavior of AO7 and identify the residual organic intermediate products at different retention times as shown in Table 2, compounds such as β-naphthol, naphthalene-1,4-dione, 4-azo benzenesulfonate, (E)-3-(2-formyl-4-hydroxy-3-nitrophenyl)-3-hydroxyacrylic acid, 2-hydroxy-8-nitronaphthalene-1,4-dione, 5-nitronaphthalene-1,4-dione were separated at t = 30 min. In addition to phthalic acid, isobenzofuran-1,3-dione, and 2-formyl benzoic acid were separated at t = 60 min. lastly salicylic acid, pyrocatechol, cyclopenta-2,4-dien-1-ol, benzenesulfonate, phenol was separated at t = 120 min. These compounds have been found as a common product that came from the degradation of AO7 in the reported literature^83–86^.

**Table 2:**
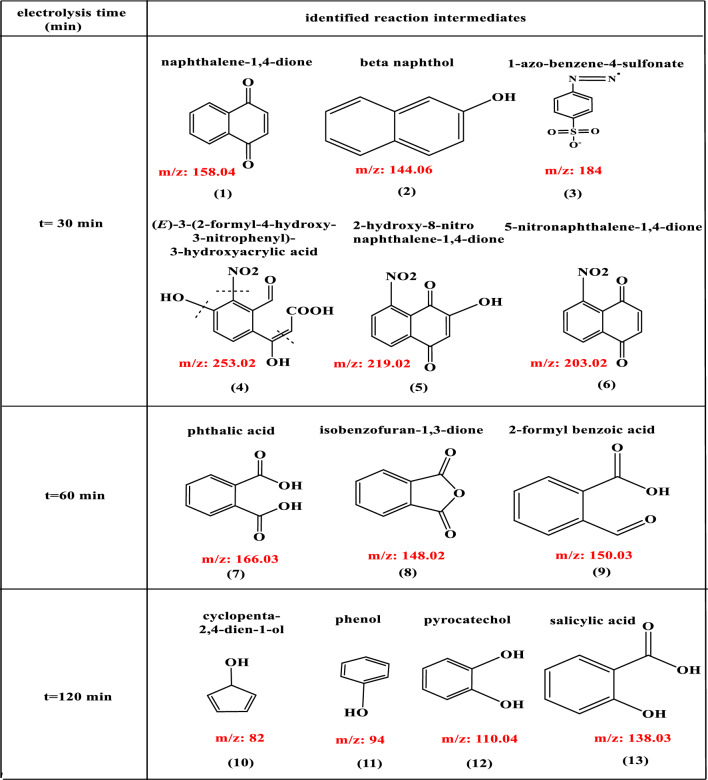
Identified reaction intermediates degradation of AO7 by GC–MS.

### Comparison with other systems

Table 3 compared the results obtained in terms of catalytic degradation efficiency, stability, reaction time, temperature, pH, and dye mineralization of AO7 in the presence of the [Co(II) TPHPP]-Cs/GO nanocomposite presented in this work, to other previously published results. It can be seen from Table 3. A heterogeneous catalyst used [Co(II) TPHPP]-Cs/GO nanocomposite gave higher degradation yield and mineralization of AO7 with a shorter reaction time required and also showed high catalytic stability and no significant changes up to the six-run compared to the other heterogeneous catalysts used in the comparison.

**Table 3:** Comparison of [Co(II) TPHPP]-Cs/GO nanocomposite with other heterogeneous systems.

Catalyst	Abbreviations	Conditions	Efficiency	References
Chitosan-capped MnO_2_-iridium nanoparticles supported on nanoceria	[Ch-MnO_2_-Ir/CeO_2_]	The oxidative degradation of acid orange 7 in an aqueous solution by activated persulfate at 30 °C, pH 9.0	The catalyst shows 80% degraded 70.06 mg/L of acid orange 7 within 100 min	^87^
Vanadium–titanium magnetite was investigated in the decolorization of Acid Orange II by a non-homogeneous Fenton process	[V-Ti]/Fe_3_O_4_	The experiments were carried out at 25 °C and the dosage of the catalyst was 0–2.0 g/L while the concentrations of AO7 and H_2_O_2_ were 0.2 mmol/L and 0–20 mmol/L	The mineralization rate was about 30% 4 h after the degradation started	^88^
Waste printed circuit board was used as a novel catalyst for the degradation of orange II with H_2_O_2_	[w-PCB]	Orange II degradation was carried out under conditions of w-PCB 2 g/L, H_2_O_2_ 5 mL/L, pH 7.9, orange II 0.1 mmol/L, and temperature 40 °C	97.98% degradation of Orange II was carried out within 6 h with (w-PCB)	^89^
Heterogeneous Fenton-like Degradation of Orange II in Water Using FeZSM-5 Zeolite Catalyst	[FeZSM-5]	The degradation of orange II performance at 266.7 mM H_2_O_2_ at 50 °C for 0.05 g/dm^3^ of [orange II] solution at a pH 7.0	A color removal of 99.99% and a COD elimination of 55.0% were achieved after a reaction time of 2 h	^21^
chitosan/MMT biocomposite in the Sono-assisted adsorption removal of AO7	[chitosan/MMT biocomposite]	The experiments were carried out under conditions of 1 g/L [chitosan/MMT], 200 mg/L [AO7], 350 W ultrasonic power, and pH 6.95	Removal efficient 51.8% within time: 60 min	^90^
Cobalt (II) complex of 5,10,15,20 tetrakis-[4-(hydroxy)phenyl] porphyrin supported on chitosan/Graphene Oxide nanocomposite	[Co(II) TPHPP]-Cs/GO	The experiments were carried out at (1.42 × 10^−4^ M) AO7 dye, with H_2_O_2_ (8 × 10^−2^ M) in the presence of [Co(II) TPHPP]-Cs/GO nanocomposite (15 × 10^−3^ g/mL) and pH = 9 at 40 °C	removal efficiency of AO7 94%, within 60 min, TOC shows 50%dye mineralization, high catalytic stability, and no significant change up to six runs	Present work

### Identification of reactive oxygen species (ROS)

#### Hydroxyl radical analysis using isopropyl alcohol

**Figure 13:**
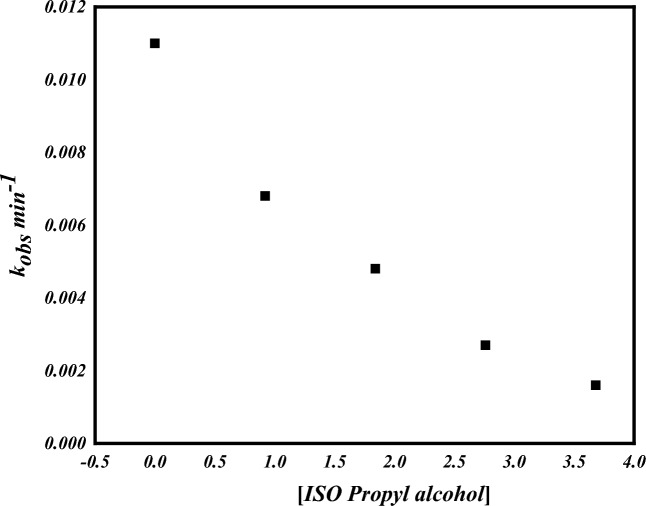
Effect of increased isopropyl alcohol on the decolorization of AO7.

Hydroxyl radicals were able to be the reactive species in the oxidation of the dye AO7 by our catalytic system [Co(II) TPHPP]**-**Cs/GO nanocomposite/H_2_O_2_, hence, the inhibiting effect of isopropyl alcohol as hydroxyl radicals scavenging agent has been investigated on the oxidation reaction of AO7 by H_2_O_2_/catalyst. As shown in (Fig. 13), the rate of degradation of AO7 decreased with the addition of isopropyl alcohol to the reaction mixture and was inhibited by increasing the concentration of isopropyl alcohol in the reaction solution. This indicates that decolorization of AO7 by [Co(II) TPHPP]**-**Cs/GO nanocomposite/H_2_O_2_ involved the formation and participation of ^•^OH radicals as the active species.

#### Hydroxyl radical determination

**Figure 14:**
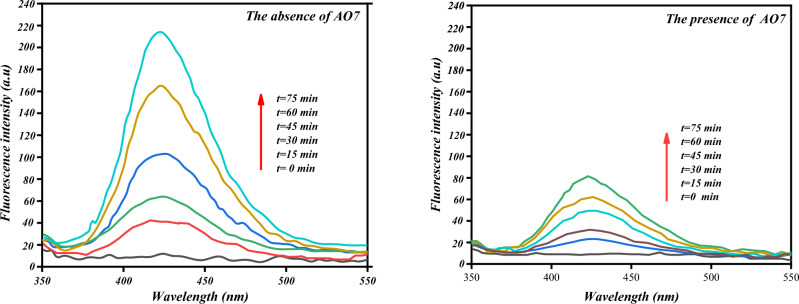
Fluorescent intensity of hydroxy terephthalic acid formed with [Co(II) TPHPP]-Cs/GO nanocomposite/H_2_O_2_, in the presence and absence of AO7.

To identify the reactive oxygen species formed in the [Co(II) TPHPP]**-**Cs/GO nanocomposite/H_2_O_2_ system, disodium salt of terephthalic acid (NaTA) photo-luminescence probing technology measurements were carried out. NaTA could react with ^**·**^OH to give 2-hydroxy terephthalic acid (HTA), which exhibits a bright stable fluorescence^91^. This reaction is unaffected by the presence of other reactive species such as H_2_O_2_, HO_2_^**·**^, and O_2_^**·**^, so it could be used as a sensitive probe in detecting ^**·**^OH radicals^92^. (Fig. 14) shows the fluorescence spectra of the solution containing the [Co(II) TPHPP]**-**Cs/GO nanocomposite/H_2_O_2_-system and NaTA.

It's observable that the fluorescence intensity increases sharply to 220 within 75 min, implying that ^•^OH radicals were indeed generated in the system. Moreover, when AO7 and NaTA were simultaneously added into the solution, the generated fluorescence significantly decreased, with the intensity only increasing to 80 simultaneously.

### Suggested mechanism

The suggested mechanism for the oxidation reaction involves an activation of the H_2_O_2_ molecule, leading to the formation of hydroxyl radicals(^•^OH). as shown in the following equations.

6 $$ {\text{S}} - [{\text{Co}}({\text{II}}){\text{TPHPP}}] + {{\text{H}}_2}{{\text{O}}_2} \to {}^\cdot {\text{OH}} + {}^- {\text{OH}} + {\text{S}} - [{\text{Co}}({\text{III}}){\text{TPHPP}}] $$

7 $$ {\text{S}} - [{\text{Co}}({\text{III}}){\text{TPHPP}}] + {}^- {\text{OH}} \to {\text{S}} - [{\text{Co}}({\text{II}}){\text{TPHPP}}({\text{OH}})] \to {\text{S}} - [{\text{Co}}({\text{II}}){\text{TPHPP}}] + {}^\cdot {\text{OH}}$$

8 $$ {\text{AO}}7 + {}^\cdot {\text{OH}} \to {\text{Residual}}\,{\text{organic}}\,{\text{intermediate}} + {{\text{H}}_2}{{\text{O}}_2} + {\text{C}}{{\text{O}}_2} $$

where S is the supported nanocomposite, [TPHPP] is the ligand, and Co is the metal ions. Cobalt can be used as a catalyst, preferably in a heterogeneous system. Cobalt is known to undergo Fenton-type reaction and has been used as an activator in the decomposition of H_2_O_2_ and the degradation of various dyes^72^. The suggested mechanism claims that the catalyst activates the H_2_O_2_ molecules, leading to the formation of hydroxyl radicals, ^**·**^OH^93^.The latter attacks the dye forming an active intermediate, which decomposes in the rate-determining step giving the final oxidation product.

### Recovery and recycling of catalyst

**Figure 15:**
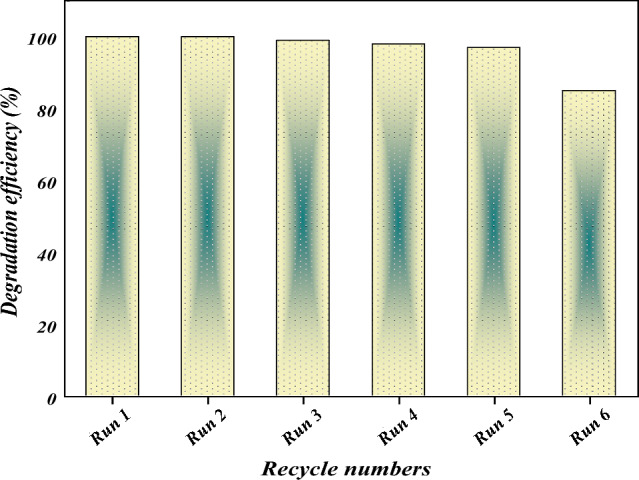
Effect of [Co(II) TPHPP]-Cs/GO nanocomposite recycling on the degradation of AO7 dye. Catalyst 15 × 10^−3^ g/mL; [AO7]: 1.42 × 10^−4^ M, [H_2_O_2_]: 8 × 10^−2^ M, pH: 9 at 40 °C.

There is a wide diversity of benefits resulting from using heterogeneous catalysts in the catalytic oxidation process as reducing reaction costs, diminishing waste generation, and bringing about more environmentally and economically saving methods for separation and recycling^24,94^. [Co(II) TPHPP]**-**Cs/GO nanocomposite was readily recovered from the solution by simple filtration and recycled for successive reactions after being rained several times with distilled water. Degradation percentages of AO7 were illustrated (Fig. 15).

The proportion of AO7 degradation after six consecutive cycles was shown in Fig. 15. No significant changes were detected for the first five successive cycles of [Co(II) TPHPP]**-**Cs/GO nanocomposite, then the foregoing catalyst losses (15%) of its activity in the sixth cycle. The results clearly showed that the mentioned supported catalyst was active in oxidative degradation and could be stable and reused without a significant decrease in activity and selectivity as shown also in FT-IR (Supplementary Fig. S11).

## Conclusions

Cobalt (II) complex of tetrakis5,10,15,20 [4-hydroxy phenyl] porphyrin-Cs/GO nanocomposite synthesized, characterized, and used to catalyze the green oxidative degradation of AO7 with hydrogen peroxide in aqueous solution. The resistive and recoverable [Co(II) TPHPP]**-**Cs/GO nanocomposite demonstrated high catalytic activity and decomposed 94% of AO7 within 60 min and showed TOC removal of 50% to CO_2_ and H_2_O with the optimum operational parameters of AO7 (1.42 × 10^−4^ M) with H_2_O_2_ (8 × 10^−2^ M) in the presence of [Co(II) TPHPP]**-**Cs/GO nanocomposite (15 × 10^−3^ g/mL) and pH = 9 at 40 °C. The breakdown rate and mineralization of the dye have been found to increase with an increase in reaction temperature and catalyst concentration up to 15 × 10^−3^ g/mL subsequently no significant increase. The rate of dye decolorization decreased with increasing the concentration of dye, H_2_O_2,_ and at higher pH than 9.0. GC–MS analyses examined all the degradation products of AO7 with different retention times. Remarkably, even after six cycles of reuse, there was no significant degradation in the catalytic activity of the recycled catalyst. This breakthrough highlights the potential of the catalyst in addressing water pollution challenges efficiently and sustainably.

### Supplementary Information

Supplementary Figures.

## Data Availability

Data are however available from the corresponding authors upon reasonable request.
