# Supplementary material for: Synthesis and characterization of Co(II) porphyrin complex supported on chitosan/graphene oxide nanocomposite for efficient green oxidation and removal of Acid Orange 7 dye
Source: Sci Rep. 2024 Jul 24;14:17073. doi: 10.1038/s41598-024-65517-z (PMC11269599; doi:10.1038/s41598-024-65517-z)
Supplement: Supplementary file 1 — Supplementary Figures. [file 41598_2024_65517_MOESM1_ESM.docx]

**Synthesis and Characterization of Co(II) Porphyrin Complex Supported on Chitosan/ Graphene Oxide Nanocomposite for Efficient Green Oxidation and Removal of Acid Orange 7 Dye**

*Sahar H. El-Khalafy^*^, Mahmoud T. Hassanein, Mohamed M. Alaskary , Nehal A. Salahuddin*

Department of Chemistry, Faculty of Science, University of Tanta, Tanta 31527, Egypt.

** Corresponding authors: Sahar H. El-Khalafy E-mail: sehar­_hasouna@science.tanta.edu.eg, Tel: +20-1007276665.*

**Supplementary Materials**

**Fig S1: Synthesis of 5, 10, 15, 20 Tetrakis [4-(hydroxy) phenyl]porphyrin [TPHPP]**

**Fig S2: Synthesis of Cobalt (II) complex of 5, 10, 15, 20 Tetrakis [4-(hydroxy) phenyl]porphyrin [Co(II) TPHPP]**

**Fig S3: Phthaloylation of chitosan**

**Fig S4: Grafting chloro-acetyl chloride on N-phthaloyl chitosan**

**Fig S5: Preparation of N-phthaloyl chitosan Supported of [Co(II) TPHPP]**

**Fig S6: Deprotection of phthaloyl group**

**Fig S7: Preparation of [Co(II) TPHPP] supported on to modified chitosan / graphene oxide nanocomposite**

**Fig S8: Electronic absorption spectra for degradation of AO7 using H_2_O_2_ (8×10^-2^M) without [Co(II) TPHPP] 1-Cs/ GO nanocomposite.**

**Fig S9: Electronic absorption spectra for degradation of AO7 using [Co(II) TPHPP] -Cs/ GO nanocomposite (15x 10^-3^g/ml) without H_2_O_2_.**

**Fig. S10: Electronic absorption spectra for degradation of AO7 using a)** **[Co(II) TPHPP] -Cs/ GO nanocomposite (15x 10^-3^g/ml) at pH=7 b) [Co(II) TPHPP] -Cs/ GO nanocomposite (15x 10^-3^g/ml) at pH=9 c) [Co(II) TPHPP] -Cs/ GO nanocomposite (15x 10^-3^g/ml) at pH=11**

**Fig. S11: FTIR for [Co(II) TPHPP] -Cs/ GO nanocomposite before and after reuse**
